# The Alzheimer’s Association Revised Criteria for Diagnosis and Staging of Alzheimer’s Disease – Where does Lewy body dementia stand?

**DOI:** 10.1002/alz.089685

**Published:** 2025-01-09

**Authors:** Carla Abdelnour

**Affiliations:** ^1^ Department of Neurology and Neurological Sciences Stanford University School of Medicine, Stanford, CA USA

## Abstract

Dementia patients often received one clinical diagnosis, yet most of these cases present multiple underlying pathologies. Bringing the transition from clinical‐based to biological‐based diagnosis holds promise with the diagnostic criteria proposed by the *Alzheimer’s Association (AA) Revised Criteria for Diagnosis and Staging of Alzheimer’s Disease* and the *Neuronal Synuclein Disease Integrated Staging System (NSD‐ISS)*. This session aims to explore the practical implications of the AA revised criteria for diagnosing and designing clinical trials in Lewy body disease (LBD).

The updated version of the AA criteria proposes 3 biomarker categories: core Alzheimer’s disease (AD) biomarkers, non‐specific biomarkers, and biomarkers for common non‐AD co‐pathologies, including synucleinopathy (asyn). Utilizing the seeding amplification assay (SAA) to detect asyn pathology expands the biomarker toolkit, historically dominated by AD biomarkers. Identifying asyn in vivo in dementia cases enables recognizing underlying LBD, potentially reducing oversight of LBD cases with concurrent AD, which are less likely to present core features (particularly females).

The AA criteria emphasize the role of clinical judgment in cases with comorbid pathology, proposing a biomarker profile for characterization. Challenges in evaluating several biomarkers in the clinical setting may be addressed using biofluid biomarkers and a stepwise process guided by clinical judgment.

Practical applications of these criteria include their role as inclusion/exclusion criteria and stratification of LBD clinical trials. Similarly, in AD clinical trials, this framework allows excluding patients with comorbid pathologies to achieve a homogeneous study population.

The AA revised criteria, along with the NSD‐ISS, serve as starting points for hypothesis testing. Future directions involve updating these frameworks with a patient‐centered approach, engaging patients and caregivers to understand the implications of biological‐based diagnosis. Longitudinal studies analyzing multiple comorbid pathologic changes are essential for determining the timeline of each pathology’s development. Finally, it is envisioned the creation of a comprehensive system to stratify disease progression of neurodegenerative diseases by incorporating biomarkers, demographic, clinical, and genetic data.

